# Potentiality of ghrelin as antioxidant and protective agent

**DOI:** 10.1080/13510002.2021.1913374

**Published:** 2021-04-14

**Authors:** Rachid Akki, Kawtar Raghay, Mohammed Errami

**Affiliations:** a Department of Plant Protection and Environment, National School of Agriculture-Meknes/ENA, Meknes, Morocco; b Department of Biology, Faculty of Sciences, Abdelmalek Essaadi University, Tetouan, Morocco

**Keywords:** Ghrelin, antioxidant, oxidative stress, systemic oxidative stress sensor

## Abstract

**Background:**

Oxidative stress is the result of cellular troubles related to aerobic metabolism. Furthermore, this stress is always associated with biological responses evoked by physical, chemical, environmental, and psychological factors. Several studies have developed many approaches of antioxidant defense to diminish the severity of many diseases. Ghrelin was originally identified from the rat stomach, and it is a potent growth hormone-releasing peptide that has pleiotropic functions.

**Methods:**

A systematic review was conducted within PubMed, ScienceDirect, MEDLINE, and Scopus databases using keywords such as ghrelin, antioxidant, oxidative stress, and systemic oxidative stress sensor.

**Results:**

In the last decade, many studies show that ghrelin exhibits protection effects against oxidative stress derived probably from its antioxidant effects. Pieces of evidence demonstrate that systemic oxidative stress increase ghrelin levels in the plasma. The expression of ghrelin and its receptor in ghrelin peripheral tissues and extensively in the central nervous system suggests that this endogenous peptide plays an important role as a systemic oxidative stress sensor

**Conclusion:**

The current evidence confirms that ghrelin and its derived peptides (Desacyl-ghrelin, obestatin) act as a protective antioxidant agent. Therefore, stressor modality, duration, and intensity are the parameters of oxidative stress that must be taken into consideration to determine the role of ghrelin, Desacyl-ghrelin, and obestatin in the regulation of cell death pathways.

## 1. Introduction

High levels reactive oxygen species (ROS), ubiquitous free radical, and other radicals are considered as harmful and physiological indicators of oxidative stress. Disproportion between ROS generation and antioxidant system leads to the onset of oxidative stress. ROS such as hydrogen peroxide, hydroxyl radical, and superoxide anion are generated continuously under physiological condition in a chain of chemical reaction during aerobic condition. However, ROS display a key role in cell function such as physiological mediators [1], in inflammation, proliferation, differentiation, aging, and other physiological functions [2].

Various physiological and pathological reactions are critically dependent to the balance in the redox system. Oxidative stress occurs as a result of redox system imbalance, which is linked to the onset of cell and tissues damage and involved in progression/propagation of a variety of pathological phenomena such as cancer [3], male infertility [4], neurodegenerative diseases [5–8], steatosis and steatohepatitis [9], pancreatic fibrosis [10], cardiac injury [11,12], diabetes [13], age-related disease and development [14].

Ghrelin a growth hormone (GH) secretagogue is now recognized as a pleiotropic and ubiquitous hormone implicated in a multitude of metabolic actions, such as appetite regulation and body weight, glucose metabolism, heart pressure, adiposity, fertility, memory, learning, and reward-related pathways [15,16]. Ghrelin binds to its cognate receptor, the growth hormone secretagogue receptor 1a (GHSR1a), identified as an essential partner for the GH release and induction of a plethora of metabolic activities. Ghrelin circulate in the bloodstream, and affect the pituitary gland releasing GH secretion. This peptide regulates energy homeostasis by transmitting information about peripheral nutritional status to the brain. Feeding behaviour is regulated by homeostatic feeding, which is dependent on energy needs, as well as hedonic feeding. Biologically active peptides as ghrelin operate inter-organ, neural, and hormonal networks and play vital roles in the control of cell-to-cell communications. The elucidation of its physiological role will contribute a better understanding of the homeostatic control system and the pathological mechanisms of various diseases. GHSR1a is widely expressed in several relevant metabolically active tissues, such as pancreas, liver, white adipose tissue, heart, gonads, and at central level in hypothalamus and pituitary gland [17]. Acyl ghrelin (AG) is the main active isoform in the cytoplasm by enzyme ghrelin O-acyltransferase (GOAT) [18], while Desacyl-ghrelin (DAG) is initially considered as an inactive form of ghrelin. However, new actions have been ascribed to DAG but the discovery of putative alternative receptor for DAG is essential to understand its novel mechanisms of action [19]. The reviewed findings reported by Satou et al. [20] regarding enzymes in the blood able of generated processed forms of ghrelin concluded that AG can be modified and cleaved in different ways that may have specific functional relevance. A clinical study about pharmacokinetics of ghrelin in young healthy subjects reported that AG disappear more rapidly from plasma than total ghrelin, with elimination half-life (*t*_1/2_) of 9–13 and 27–31 min, respectively, reflecting the rapid degradation of AG. Both the low and high doses of ghrelin strongly stimulated GH release (peak plasma concentration (*C*_max_,0–90 min): 124.2^63.9 and 153.2^52.2 ng/ml for 1 and 5 mg/kg ghrelin, respectively). However, insignificant alterations of blood glucose and insulin levels after the injection were observed [21].

Even though, ghrelin is a gastric hormone, many studies have recently reported that ghrelin reduce the inflammation processes by promoting a strong inhibition of inflammatory cytokine expression and attenuating the oxidative stress in different organs [22]. Obestatin a derived peptide from preproghrelin as well seems to exert antioxidant and antiapoptotic effects. The aim of this overview is to provide the last evidences that ghrelin exerts antioxidant effects by preventing the peroxidation and enhancing the activity of antioxidant enzymes in several tissues and organs.

## 2. Redox system and physiopathology of oxidative stress

Redox system plays an important role in physiology and physiopathology of organs and tissue, where the increased production of ROS results on oxidative stress in different tissues. Since its discovery in 1894, ROS and reactive nitrogen species (RNS) were considered as harmful agents for the cell and its components [3]. However, in the last thirty years, a substantial body of evidence suggested that ROS and RNS also function as physiological mediators [8].

Indeed, at high concentrations, those oxidant agents harm proteins, DNA, lipids, and carbohydrates resulting on oxidative damage which leads to different pathological conditions. However, at physiological concentration, they function as second messengers, playing an important role in cell regulation and signal transduction of multiple physiological processes [8,23].

Organisms have developed effective protective strategies against oxidative damage through cellular mechanisms to reduce the pathological situation responsible for deleterious processes in the cell. These strategies include endogenous enzymatic antioxidants defense and non-enzymatic molecules [3,23]. The non-enzymatic molecules are either natural substances obtained from daily nutrition or synthesized by the body itself. The non-enzymatic antioxidant function through direct removing of free radical intermediates, and/or indirectly through regulation of vital endogenous antioxidants enzymes, and/or transcription factors [24].

The alteration of ROS generation and their eliminators may be an important factor to determine cell fate. In fact, attenuation of ROS production or elevation of ROS scavengers may reduce ROS signaling, lead to impairment of ROS-mediating signal transduction [25].

ROS generated by different hormones and growth factors have been shown to affect signal transduction pathways in various cell types and may possess distinct biological functions. For instance, nicotinamide adenine dinucleotide phosphate oxidases, (NOXs)-mediated generation of ROS functions as an essential signal in the insulin-stimulated signaling transduction under normal condition [3]. Goldstein et al. reported that insulin-stimulated H_2_O_2_ modulates proximal and distal insulin signaling, at least in part through the oxidative inhibition of protein tyrosine phosphatases (PTPases) that negatively regulate the insulin action pathway. In addition, Nox4 as a homolog in the family of nicotinamide adenine dinucleotide phosphate oxidase catalytic subunits was expressed in insulin-sensitive cells. This molecule was shown to mediate insulin-stimulated H_2_O_2_ generation and impact the insulin signaling cascade. Overexpression of Nox4 also significantly reversed the inhibition of insulin-stimulated receptor tyrosine phosphorylation by PTP1B involved in the negative regulation of insulin signaling, by inhibiting its catalytic activity [25].

Consequently, the role of antioxidants acting as the first line defense to eliminate ROS effects may provide highly efficient manner to maintain cell integrity in response to stress stimulus.

## 3. Ghrelin strategies in reducing oxidative stress processes

The reduction of oxidative stress has long been viewed because of slowing metabolism and activating the control of the defense program. Shreds of evidence provide that oxidative stress is resulted from physical as psychological stress [26]. In fact, significant studies have demonstrated that protein oxidation is related to the expression of a variety of human diseases as diabetes, atherosclerosis, fertility, and neurodegenerative disorders [27–29]. Many antioxidant intervention strategies are available in the organism but currently, new peptides as antioxidants have appeared. Their ability to inhibit lipid oxidation through multiple pathways including inactivation of ROS, scavenging free radicals, chelation of prooxidative transition metals, and reduction of hydroperoxides which makes them more interesting in oxidative stress and improving defence of biological tissues. Ghrelin, the second most abundant gastric endocrine cell type is secreted by X/A cells [16]. This hormone of 28 amino acids is involved in a variety of physiological and pathological processes. Previous findings demonstrated that ghrelin is an endogenous antioxidant and functions as a free radical scavenger [30]. It has been demonstrated that ghrelin inhibits apoptosis by down-regulation of Bax, preventing cytochrome c release, and inhibition of ROS formation, increasing antioxidant enzyme activities, and reducing lipid peroxidation [31]. In peptide biology, many peptides could operate in isolation versus interdependently, locally, or systemically. Previously, we reported that ghrelin (acylated or unacylated) has an anti-inflammatory and protective effect on ischemia–reperfusion injury of diverse organs [22], which would be an excellent candidate as a sensor of systemic oxidative stress. Recently, using primary cultures of mouse gastric mucosal cells in combination with three well-known antioxidants (resveratrol, SRT1720, curcumin) provided that lowering oxidative stress within ghrelin cells stimulates ghrelin secretion through activation of Nuclear factor erythroid-derived-2-like 2 (Nrf2) a master antioxidative response regulator which blocks the effect of glucose to inhibit ghrelin secretion [32]. However, further studies should be directed to investigate how alterations of redox state within ghrelin cells acting under different physiological settings.

### 3.1. Antioxidant and protective effect related to CNS

Ghrelin is secreted by the stomach and reaches the brain by crossing the blood–brain barrier and transmits its signal through the vagal nerve [33,34]. Normal adult plasma samples contain 100–120 fmol ghrelin per ml, indicating that it is not secreted in the gastrointestinal tract, but into the systemic circulation, exhibiting endocrine, paracrine, and possibly autocrine effects [35].

Oxidative stress plays a key role in the development of various neuropathological diseases, such as epilepsy [36], ischemia, Parkinson’s disease. In a rat model of epileptic seizures, Obay et al. have reported that intraperitoneal injections of ghrelin can protect against the development of epileptic seizures induced by pentylenetetrazole (PTZ) [37]. The same research group consequently has demonstrated that ghrelin greatly prevents or delayed PTZ-induced neuron damages in a dose-dependent manner by preventing the reduction of antioxidant enzyme activities and abolished oxidative stress. The authors concluded that this could be the possible mechanism by which ghrelin injection induces anticonvulsant effect [38]. Interestingly, DAG significantly elevates seizure thresholds in C57Bl/6 and wild-type mice but not in ghrelin receptor knockout mice suggesting that DAG exert anticonvulsant effect via ghrelin receptor rather than orexin pathway [39]. Those evidence has shown that ghrelin exerts neuroprotective and anti-inflammatory effects on neuron from epilepsy-induced damage [36].

In another study using ghrelin knockout mice, it was reported that endogenous ghrelin suppressed superoxide overproduction and regulates the vasodilator effects of nitric oxide (NO) in male mouse cerebral circulation [40]. Furthermore, ghrelin administration improves cerebral blood antioxidant status in the brain under hypoxic condition via the reduction of blood malondialdehyde (MDA) levels that is an indicator of increased oxidative stress [41]

Preconditioning ascribed as an endogenous protective mechanism induced protection of neurons against ischemia/reperfusion injury [8,42]. Ischemic tolerance was associated with a variety of signaling pathways activated by preconditioning, including increased antioxidant activity and reduction of ROS production [23]. Liu et al. have been demonstrated that both administrations of ghrelin that possesses protective and antioxidant properties and ischemic preconditioning protect hippocampus CA1 neurons against ischemia–reperfusion induced ROS overproduction via increased expression of the mitochondrial uncoupling protein UCP2 [42].

Under hypoxic conditions, ghrelin treatment was described as a protective agent of neuronal cell death induced by increased oxidative stress through increasing total antioxidant capacity and reducing MDA levels [41].

In a preclinical study, Seyhanli and colleagues have been shown that acute ischemic stroke patient’s induced oxidative stress was associated with the alteration of AG and DAG levels. DAG was elevated in patient serum with acute stroke, but AG levels were increased in patient urine with acute stroke [19]. In addition, the previous study has observed alteration of ghrelin expression in patients with high-risk factors like obesity, diabetes, and aging [43]. These findings indicate that urine AG levels may be considered as a diagnostic parameter in acute ischemic stroke patients and further studies are needed to support this finding.

Concerning the spinal cord, ghrelin has been reported to possess a neuroprotective activity. Apparently, the oligodendrocytes are very sensitive to elevated ROS production due to their low capacity of antioxidant status and other intrinsic risk factors [44]. Therefore, the salvation of oligodendrocytes under oxidative stress condition is mediated by ghrelin treatment. This treatment promotes oligodendrocyte survival via up-regulation of ERK signaling and down-regulation of p38MAPK after treatment with hydrogen peroxide-induced spinal cord oxidative damage [44]. Furthermore, ghrelin antiapoptotic effect mediated by ghrelin receptor was described. Therefore, targeting ERK signaling pathway as a pharmacological target of natural phenolic compounds improves neuronal performance and prevents or delays the onset of major neurodegenerative disease [45].

### 3.2. Antioxidant and protective effect related to the gastrointestinal tract

Most gastrointestinal damages are triggered by physical or psychological stress. In response to oxidative stress, the activity of the hypothalamic–pituitary–adrenal axis is increased to counter the harmful effects of gastric ulceration. Suzuki and coworkers have reported the association of oxidative gastrointestinal damages with the potential protective role of ghrelin [26]. Previous studies have shown that ghrelin exhibits gastroprotective effects such ghrelin effects were mediated by endogenous NO release via sensory nerve fibers [46]. It was hypothesized that the gastroprotective effect of ghrelin central administration ensures an adequate amount of constitutive derived NO and prostaglandin (PGE_2_) by enhancing eNOS mRNA expression. However, the peripheral effect of ghrelin remains unresolved before considering ghrelin as a potential anti-ulcer drug [46]. Oxidative stress induces not only gastric mucosal injury but also gastric motility dysfunction called gastroparesis. Clinical trials conducted by using a synthetic ghrelin agonist (TZP-101/Ulimorelin), demonstrated that this prokinetic agent accelerates changes in the plasma ghrelin levels, improving gastric empty and meal-related symptoms in diabetic patients. Furthermore, TZP-10/ulimorelin treatment accelerates gastrointestinal motility recovery in patients undergoing surgery [47,48].

Generation of large amounts of ROS during the pathogenesis of gastric mucosal injury is the most known stress induced by excessive ingestion of non-steroidal-anti-inflammatory drugs. Evidence showed that ghrelin administration after treatment with sodium metabisulfite (Na_2_S_2_O_5_) decreases gastric total oxidative status depleting antioxidants levels in gastric mucosa of rats. Furthermore, ghrelin treatment significantly demonstrated a decrease in the number of apoptotic cells while Ki67 expression is increased in gastric mucosa exposed to Na_2_S_2_O_5_ [49].

Different strategies have been tested to reduce ROS overproduction, which induces serious clinical repercussions involved in pathologies, surgical interventions, or severe treatment like cancer chemotherapy. These factors are responsible to induce systemic oxidative stress enhancing the plasma ghrelin levels and inducing the expression of antioxidant enzymes [26].

The potential protective and therapeutic effects of ghrelin on oral mucositis were reported. The concentrations of ghrelin in saliva are similar or higher than plasma, a decreased saliva secretion in patients under chemotherapy or radiology makes them prone to develop oral mucositis [50]. Furthermore, experimental studies performed on rats have shown that intraperitoneal administration of ghrelin accelerated the healing of oral ulcers. In addition, clinical data also provide that ghrelin level in the gingival cerevicular fluid is lower in the patients affected by periodontitis than healthy subjects [51]. We suggest that intraperitoneal injection of ghrelin could act on peripheral targets by binding to GHS1aR and stimulating a cascade of protective signals, helping to decrease lipid peroxidation and reduce the expression of ROS in response to oxidative stress induced by pathological settings.

Beneficial ghrelin effect on the healing of colonic anastomosis in rats was studied [52]. The increase in the tissue concentration of ROS on the third postoperative day returned to basal levels on the 14th day, demonstrating the effectiveness of the antioxidants system. The recovery and maintenance of redox homeostasis promote optimization of the healing process.

### 3.3. Antioxidant and protective effect related to hepatic tissue

In liver pathologies such as Nonalcoholic fatty liver disease (NAFLD) is considered the main insult responsible for lipotoxicity characterized by severe oxidative stress which is involved in insulin resistance (IR), lipid peroxidation, mitochondrial deregulation, and liver chronic inflammation and apoptosis [9]. The progression key of these damages is oxidative stress and inflammation. In fact, the most significant risk factor to develop NAFLD is the high free fatty acids in obese and diabetic patient circulation [9]. Therefore, NAFLD is a progressive disease that can reach the hepatocellular carcinoma stage in the most complex cases. Previous studies have reported that ghrelin–ghrelin O-acyltransferase (GOAT) system is involved in the pathogenesis of NAFLD ([53]; Quiñones et al., 2020 [106]). The acylation of the ghrelin is catalyzed by GOAT during the processing peptide [9,18]. Indeed, acylated ghrelin as a supplement for obese animal diet was able to normalize redox state and inflammation markers by limiting fat accumulation in the liver [54]. More evidence has been reported by Koyoturk and colleagues, on the role of AG in the liver of neonatal diabetic rats. In responses to subcutaneous administration of ghrelin, the antioxidant enzymes including glutathione, catalase, and superoxide dismutase activities were increased. It is well known that GSH is predominantly synthesized in the liver, and it is considered the most abundant intracellular antioxidant in this organ. Outcomes provide that ghrelin administration improves the GSH depletion in neonatal diabetic rats, which would impair the defense against oxidative stress [55].

In previous studies, long-term administration of ghrelin in chronic liver injury and fibrogenesis rat models induced by thioacetamide, revealed that ghrelin reduced hepatic injury by a partial protective effect attributed to increased NO levels [56]. However, the role of NO and its beneficial effect on these pathologies is still inconclusive, because small amounts of NO may exert a cytoprotective effect, while overproduction of NO may damage liver function. The decrease in the production of MDA and NO-induced by ghrelin pretreatment showed that the anti-lipid peroxidation potency of ghrelin is involved in liver protection [57]. As known, activated macrophages are the main source of peroxynitrite generation leading to nitrotyrosine formation [58]. Remarkably, during cardiopulmonary bypass (CPB) on rats, ghrelin pretreatment was able to prevent macrophage infiltration by reducing tyrosine nitration in the lung and attenuating by half in the liver [59]. Therefore, these findings suggest an improvement by ghrelin pretreatment suppressing the activation of the macrophage infiltration contributing thus to reducing the elevation of oxidative stress.

The protective role played by ghrelin against paracetamol-induced hepatotoxicity and carbon tetrachloride (CCl4)-induced acute liver injury in rats was demonstrated by evaluation of lipid peroxidation, enzymes activities, and biochemical parameters [57,60]. Results revealed that ghrelin pretreatment reduced plasma and liver MDA content, plasma NO level, and increased liver tissue SOD, MnSOD, CAT, and GPx activities compared with the control group [57,60,61]. This decline in MDA may be attributed to oxidative inactivation of enzyme protein by excess ROS generation [57], while the protective effect may depend on the inducible effect of hemeoxygenease-1 (HO-1) that provides more cytoprotection against oxidative stress in a variety of experimental models [60]. Furthermore, the ERK1/2 and Akt signaling pathways were involved in ghrelin-mediated regulation, of the protein expression of antioxidant enzymes and iNOS in the rat liver [61].

Interestingly, the impact of DAG on oxidative stress and inflammation was reported in the liver of diabetic rats [62]. In this study, the administration of DAG in a diabetic animal model counteracts all diabetogenic effects of streptozotocin. DAG reduces lipid peroxidation levels by a significant decrease in MDA formation and increases on SOD and GPx activities to control the imbalance between ROS production and their removal.

### 3.4. Antioxidant and protective effect related to pancreatic tissue

Other evidence provides that ghrelin has a protective effect on pathogenesis associated with the pancreas, such as pancreatic fibrosis [10] and acute pancreatitis (AP) [63,64]. Ghrelin was reported as a protective hormone against the development of AP by activation of capsaicin-sensitive sensory nerves [63,64]. This protection was correlated with the release of growth hormone (GH) and insulin-like growth factor-1 (IGF-1). While pancreatic fibrosis was induced by activation of mTORC1 signaling in gastric X/A-like cells and subsequently impairs glucose homeostasis through ghrelin suppressing [10]. Inhibition of this signaling pathway by exogenous ghrelin administration may thus offer an alternative strategy for the therapy of pancreatic fibrosis and its related disorders such as diabetes.

The protective effect of ghrelin was also demonstrated in the pancreas. Several In *in vivo* and *in vitro* models of AP have shown that pretreatment with ghrelin stimulates partial protection against AP development induced by cerulein. This effect was abolished when acinar cells isolated from rats with capsaicin-sensitive sensory nerves ablation. Furthermore, this result shows that the conservation of sensory nerve integrity is mandatory for ghrelin protective effect in the pancreas [65]. Similar protective effects of ghrelin were also observed against pancreatic damage induced by ischemia–reperfusion [66].

Endocrine Metabolic disorders as obesity are considered the primary metabolic abnormality leading to hepatic lipid accumulation consist of alterations in the uptake, synthesis, degradation, or secretion of lipid molecules, resulting from hyperinsulinemia and IR [15]. Obesity is strongly associated with the increase of oxidative stress as a result of the imbalance between antioxidants and pro-oxidants [67]. Studies realized in obese children have shown that ghrelin plasma levels are increased compared to normal subjects. Insulin and ghrelin are two hormones involved in the regulation of body weight and are associated with the induction of insulin secretion and oxidative stress [68]. Other evidence has associated AG and obestatin with IR where ghrelin levels were decreased in obese subjects [69]. Studies in obese children have shown a high level of ROS accompanied by a reduction in antioxidant status [67]. Ghrelin level in obese subjects was negatively correlated with body mass index and MDA levels, thus may be considered a suitable target for the management of IR [69].

Novel treatments focusing on obesity are being investigated. In humans, immunization against ghrelin had entered Phase I and IIa trials as an anti-obesity therapy. In a randomized, double-blind, and placebo-controlled trial with 87 obese patients aged 18–55 years with a body mass index between 30 and 35. Participants received four injections of 300 µg of the vaccine or placebo at weeks 0, 4, 8, and 16, despite a strong response in ghrelin autoantibodies. The median weight loss was 3.6 kg after 6 months in both groups, but the treatment was safe and well tolerated [70]. The anti-ghrelin vaccine decreases food intake, decreases hypothalamic orexigenic signals, and increases energy expenditure, at least in rodents and pigs. Therefore, therapeutic vaccines using anti-ghrelin antibodies are still not available as an alternative treatment tool to be used with diet and exercise to treat obesity.

### 3.5. Antioxidant and protective effect related to male reproductive system

The most common abnormalities related to male infertility reduced semen quality [71], lower sperm count [72,73], decreased sperms motility as well as increased DNA fragmentation index [71]. Basically, two main factors contribute to male infertility in obese animals: oxidative stress and hormonal changes. These variations are associated with endocrine abnormalities which were observed in overweight and obesity inducing lower plasma level of follicle-stimulating hormone and luteinizing hormone [4], lower total free testosterone, and increased DNA fragmentation index, while oxidative stress could play an important role to understand the relationship between the increased DNA damage in sperm of overweight or/and obese men. In testis, ghrelin was localized mainly in Leydig cells and in a lesser extent in Sertoli cells, which confirm ghrelin role in the control of spermatogenesis. In addition, studies have shown that the onset of puberty has been delayed by ghrelin in male rats and regulates Leydig cells proliferation [74,75]. Evidence provides that chronic administration of ghrelin supports the antioxidative defense system in the rat testis by the increase of GPx activity and reduction of MDA level [76].

Exercise induces full protection against sperm abnormality and changes in the level of semen parameters, sexual hormone by up-regulating Stem Cell Factor, and modulating lipid profile through correcting sperm deformities by down-regulating ghrelin levels in testis [4]. Previously, many studies have been demonstrated that regular exercise induces protection, which is partially attributed to increased antioxidant system activities and decreased oxidative stress levels [77,78]. Beside its regulation effect on reversing ghrelin levels and hormonal imbalance, endurance exercise, on the other hand, provide more protection in testis by boosting antioxidant parameters [4].

Previous studies suggest that germ cells are the most susceptible cells to apoptosis induced by elevated temperature that leads to cell death [79]. Hyperthermia induced cell death by increased ROS production [31], up-regulation of Bax expression, and the release of cytochrome c [80,81]. However, it has been demonstrated that ghrelin treatment reverses testicular damage promoted by hyperthermia through decreasing ROS generation, down-regulating of Bax expression, in turn abolishing cytochrome c release [82], and increasing thus antioxidant enzyme activities [31].

Moreover, treatment with ghrelin and its agonists prevent or minimize testicular damage that occurs after treatment with chemotherapeutic drug cisplatin-induced gonadal toxicity in cancer patients [83]. These beneficial effects of ghrelin on human testis were associated with its antioxidant activities [84].

Interestingly, in male rat models of metabolic syndrome that was associated with hypogonadism in humans, administration of ghrelin modulated the negative effects of fructose-enriched diet on sperm quality, by an increase in Gpx3 expression in the epididymis. However, ghrelin did not improve the metabolism of fructose-enriched diet animals and failed to reverse hypogonadism [85].

### 3.6. Antioxidant and protective effect related to renal system

In a mice model of renal damages, Fujimura et al. [86] demonstrated that treatment with exogenous gut peptide ghrelin induced renal protection through antioxidative mechanisms. The antioxidative effects of ghrelin were mediated by increased expression of mitochondrial uncoupling protein (UCP2) and Peroxisome proliferator-activated receptor γ co-activator 1 α (PGC1α) expression. Ghrelin exerts a decrease in mitochondrial ROS levels by reducing mitochondrial membrane potential and inhibiting excessive ATP generation [86]. In addition, the renal dysfunctions as fibrosis or senescence changes related to oxidative stress were regulated by the endogenous ghrelin/GHSR1a pathway. Ghrelin pathways display a vital role against the onset of renal premature senescence and the progression of kidney cell death induced by angiotensin II. The up-regulation of UCP2 by ghrelin through activation of AMP kinase in mitochondria confirm the protective effects of ghrelin by reducing ROS generation, which provides evidence that endogenous ghrelin plays an antioxidative role in maintaining the renal redox status [86].

In the rat renovascular hypertension model, ghrelin exhibited an anti-hypertensive effect via regulation of oxidative stress resulted through renin-angiotensin system (RAS) dysfunction. The antioxidative stress effect of ghrelin was due to its reverse action on plasma renin activity (PRA) and angiotensin II receptor type 1 (AT1R). Ghrelin actions were elucidated through decreasing PRA levels and down-regulating angiotensin II receptor type 1 [87].

In the ischemic acute renal failure mice model, ghrelin improved the vascular endothelial function and renal excretory function, which result in preventing renal damage via the insulin growth factor-1 (IGF-1) signaling pathway. The increase of IGF-1 by ghrelin provides a protective effect in renal function [88].

Moreover, other studies report ghrelin kidney protective effects through its antioxidant properties. These results demonstrated that treatment with exogenous ghrelin alleviated oxidative damage via decreased lipid peroxidation [87] and subsequently elevated the activity of antioxidant enzymes, such as GPx, CAT, SOD, and GSH content on rat kidney [89–91].

### 3.7. Antioxidant strategies of ghrelin in age-related diseases

Aging is the gradual loss of homeostatic mechanisms associated with an accumulation of molecular oxidative damage. Oxidative stress promotes the pathogenesis of several cardiovascular, pulmonary, and neuronal disorders in elderly people. Amitani et al. (2017) have reported and discussed aging and its relationship with ghrelin [107]. Concluded evidence exposed that caloric restriction mimetics and ghrelin mimetics may provide new hope for improving a healthy life in elderly persons. However, in a clinical study, a set of the clinical biomarkers (plasma ghrelin, insulin, leptin, interleukin 6, adiponectin, testosterone) was investigated to distinguish between long-lived (older than 90 years) and short-lived (72–76 years of age). Results demonstrated that none of the single biomarker levels was significantly different between the two groups, which suggests that a combination of multiple biomarkers is needed to be an effective biomarker of health [92]. Moreover, the efficacy regarding longevity remains controversial and many clinical studies are needed to clarify the mechanism of ghrelin involved in longevity.

Many factors contribute to progressive retinal ganglion cells (RGCs) death such as age-related macular degeneration, cataract, glaucoma [93]. The expression of ghrelin has been shown in rat retina and other structures of the eye, especially in retina endothelial cells, posterior epithelium of the iris, in retinal Müller cells, and choroid cells [94,95]. The ghrelin expression in the retina may suggest its involvement in the physiology and physiopathology of this photoreceptor structure [96].

Glaucoma inducing vision loss occurs as a result of a progressive increased ocular pressure, which in its turn induced retinal ganglion cells (RGCs) degeneration [93,97]. Ghrelin administration was associated with a decreased ocular hypertension [98]. Moreover, ghrelin treatment reverses the effect of glaucoma by decreased the levels of NO synthase-2 (NOS_2_) and MDA in the anterior ocular chamber fluid [93]. In addition, the ghrelin neuroprotection effect was reached in retina by up-regulation of AKT-mTOR signaling pathway [99], decreasing thus ROS damage and restoring the mitochondrial function [99,100].

In an *in vitro* study using human retinal microvascular endothelial cells (RMECs), DAG pretreatment has been shown to block apoptosis induced by oxidative stress, through up-regulation of SIRT1 signaling pathway and its downstream targets antioxidants enzymes like CAT and MnSOD [101]. The retinal neuroprotection effects of ghrelin were elicited through its antioxidant activity, which was mediated by inhibiting the increment of apoptosis [99,100], autophagy, and partially via decreasing retinal Müller cell gliosis [96].

CPB contributes to the development of postoperative complications, including respiratory failure, myocardial, renal, and neurological dysfunction, and ultimately can lead to the failure of multiple organs. ghrelin pretreatment attenuated the local oxidative stress by sustaining plasma levels of reduced glutathione and decreased glutathione disulfide in rat CPB model [59].

Another finding supports the beneficial effects of DAG on the vascular function and early atherosclerosis development associated with obesity. DAG treatment reduced vascular oxidative stress, resulting in the prevention of early atherosclerosis and lipid accumulation in the vascular wall. Those effects were independent of eNOS pathway [102].

Accumulating evidence indicates that ghrelin plays a central role in the neurohormonal regulation of food intake and energy homeostasis. Ghrelin affects both endocrine and exocrine glands and has a stimulatory effect on the secretion of most hormones of the endocrine system [103]. In a study in PD patients, ghrelin was assessed in the early stages of the disease and plasma ghrelin levels were decreased. However, this event might be irrelevant to PD progression [104]. The available *in vivo* study evidence suggests that ghrelin has either a null or inverse association with risk or progression of most cancers, although there is not enough evidence to confirm that this holds for all cancers [105]. Further clinical trials are necessary to demonstrate the association between ghrelin levels and early development of pathological events.

## 4. Conclusions

This present overview suggests by these interesting data that point on ghrelin potential as an antioxidant agent in many pathologies and Age-Related Diseases. Ghrelin also adopts strategies to provide significant protection in many organs. However, it remains to be determined how DAG which is the predominant form in circulation, is unable to bind but to activate GHSR1a [39].

Technological advances in ROS detection and quantification among others will be beneficial to better understanding the involvement of endogenous hormones like ghrelin and its derived peptides in redox signaling. Finally, further studies and investigation of ghrelin role in the oxidative process may facilitate the development of targeted therapeutic antioxidant strategies.
